# Valuing SF-6Dv2 Using a Discrete Choice Experiment in a General Population in Quebec, Canada

**DOI:** 10.34172/ijhpm.8404

**Published:** 2024-09-07

**Authors:** Hosein Ameri, Thomas G. Poder

**Affiliations:** ^1^School of Public Health, University of Montreal, Montreal, QC, Canada.; ^2^Centre de Recherche de l’IUSMM, CIUSSS de l’Est de l’Île de Montréal, Montreal, QC, Canada.

**Keywords:** SF-6Dv2, Health Utilities, Valuation, Discrete-Choice Experiments

## Abstract

**Background:**

An updated version of the Short-Form 6-Dimension (SF-6D) Classification System has been developed. This new version (SF-6Dv2) with improved consistency and dimension descriptors is now requiring the development of new utility value sets. The aim of this study was to estimate an SF-6Dv2 value set from a general population in Quebec, Canada.

**Methods:**

A discrete choice experiment with time trade-off (DCE_TTO_) was conducted using two designs: binary choice sets (Design 1) and best-worst choice sets (Design 2). Design 1 consisted of binary choice sets along with an associated duration, and Design 2 included Design 1 and a third scenario describing "immediate death." Various logit model specifications were employed to estimate value sets separately for Design 1 and in combination with Design 2. Heterogeneity in preferences was assessed using a mixed logit model.

**Results:**

The survey was completed online by 1208 participants and 1153 were included for analysis. The model combining Design 1 and 2 data was considered as the best fitting model for estimating the final value set. It provided a value set with logical consistent coefficients and showed the lowest standard errors. Values ranged from -0.683 for the worst health state (555655) to 1 for full health (111111), with 13.01% of the values being negative. Preference values were the most affected by pain dimension and the least by vitality dimension. Preference heterogeneity existed for all the most severe levels of dimensions.

**Conclusion:**

This study provided the SF-6Dv2 value set for use in Quebec, Canada. The recommended value set is the anchored consistent model combining data from Design 1 and 2 using a conditional logit.

## Background

**Key Messages**

**Implications for policy makers**

- Various health technology assessment (HTA) agencies recommend using a value set specific to the preferences of the population targeted to calculate quality-adjusted life-year (QALY).
- This study provides a value set for the Short-Form 6-Dimension version 2 (SF-6Dv2) from the preferences of the general population in Quebec, Canada.
- Two designs were used to gather data from a general population in Quebec, and the model that combines both designs is considered as the best fitting one.

**Implications for the public**

Cost-utility analysis is the method recommended by health technology assessment (HTA) agencies to estimate the efficiency of healthcare programmes or technologies. Utility is measured with quality-adjusted life-year (QALY) instruments. The Short-Form 6-Dimension version 2 (SF-6Dv2) is one of the most popular instruments. It is important that the value set used to calculate QALY corresponds to the values and preferences of the society to which it refers. If not, there is a risk for the value set to under or over-estimate the change in QALY since an inappropriate value set will provide different weights to the health dimensions used in the measurement of health-related quality of life (HRQoL). This study eliminated this risk by producing a value set specific to the preferences of the Quebec population. In addition, this study used the combination of two designs to provide more efficient and consistent results.

Countries with publicly funded health services often face with challenges when trying to achieve universal health coverage.^1^ The decision-making process regarding the allocation of resources to healthcare technologies and interventions has thus gained paramount importance. As a result, there is an interest in health technology assessment (HTA) due to the crucial role of economic evaluation.^2,3^ Although economic evaluation can be conducted using natural outcomes such as life-years saved, it is important to recognize their limitations in capturing only one aspect of health (eg, mortality). Quality-adjusted life-years (QALYs), which combine length of life and health-related quality of life (HRQoL) in a single index between 0 (death) and 1 (full health), are frequently recommended as the unit of health outcome in economic evaluation guidelines of countries such as Canada.^4,5^ QALYs are mostly calculated using generic preference-based measures such as the EuroQol Five-Dimension (EQ-5D) and the Short-Form 6-Dimension (SF-6D).

 The SF-6D is one of the most common indirect instruments, which was developed based on general health dimensions, and can be used in both health services research and clinical settings.^6^ This instrument includes a descriptive system, and a utility value set that can be applied to generate utility values. The SF-6D can be used directly in two versions: SF-6D version 1 (SF-6Dv1) and SF-6D version 2 (SF-6Dv2).^7^ The SF-6Dv1 is constructed from a set of 11 items sourced from the 36-item Short-Form survey (SF-36) and its descriptive system includes six dimensions each with four to six levels: physical functioning (PF, 6 Levels), pain (PA, 6 Levels), social functioning (SF, 5 Levels), role limitation (RL, 4 Levels), mental health (MH, 5 Levels), and vitality (VT, 5 Levels). Hence, this instrument defines a total number of 5^3^*6^2^*4 = 18 000 distinct health states.^6^ Another component of the SF-6Dv1 is the value set, which is derived by assessing the preferences of a representative sample of the community on the health states derived from the instrument’s descriptive system. A number of countries have generated country-specific value sets for the SF-6Dv1 using methods like standard gamble, time trade-off (TTO), and discrete choice experiment with time trade-off (DCE_TTO_).^8^

 Nevertheless, there is evidence indicating limitations of the SF-6Dv1, including ambiguity in differentiating between intermediate severity levels within the PF dimension, a positive framing bias observed in the VT items when compared to the other dimensions, the lack sensitivity of the RL dimension, and the tendency to generate high values, particularly for severe health states. To overcome these limitations, a new version of the SF-6D (ie, SF-6Dv2) was recently developed by Brazier et al in 2020.^7^

 The SF-6Dv2 is an improved version of the SF-6Dv1 in terms of number of dimension levels and rephrased items, while its descriptive system definitions remain the same as SF-6Dv1. This version is derived from 10 items of the SF-36, and range of responses in SF-6Dv2 has been expanded to 5 to 6 levels, thereby generating a total of 18 750 possible unique health states (5^5^*6^1^).^7^ In addition, the RL has been modified to provide more details, the PA description has been altered to focus on pain severity instead of frequency, the MH description has been updated to align with SF-36 wording, and the VT has been adjusted to include negative wording.^9^ To date, four countries, including the United Kingdom,^10^ Australia,^11^ China,^12^ and Iran^13^ have developed local value sets for the SF-6Dv2 based on the perspective of a representative sample of their general population. In Canada, specific value sets for the SF-6Dv2 have been derived for two distinct groups of diseases, namely cancer^14^ and food allergy.^15^ These value sets are designed for use in individuals with these specific diseases, while value sets are often used in health policy decision-making processes where considerations extend beyond the perspectives of individual patients. In such cases, it is important to capture societal values and preferences in order to inform resource allocation and policy choices effectively.^16^ However, the use of alternative value sets is limited due to disparities in culture, economy, or other socioeconomic factors across countries, even when employing the same standard survey procedure and similar modeling techniques.^17^ Therefore, having a specific value set to each group or country is a more suitable approach. So far, no Canadian value set for the SF-6Dv2 from the general public’s perspective has been available for the calculation of QALYs. This study aimed to generate a value set for SF-6Dv2 from the perspective of the general population in Quebec, Canada. Quebec is known to be a distinct society within Canada, with strong cultural values, thus justifying conducting a study in this single province.

## Methods

###  Study Population

 We recruited a representative sample of the general population from the province of Quebec, Canada, where French is the first language for a majority with 95% of the population able to read and speak in French, through an online panel managed by Survey Sampling International. The survey was conducted in 2016 and 2018 using a quota sampling technique and only French-speaking adults living in Quebec were randomly invited to participate. The survey was initially designed to compare the standard gamble method with the DCE method in eliciting QALYs values. Participants decided whether to join and were given a unique ID to prevent duplicates. Only those who completed the entire survey, including the EQ-5D-5L section, were included in the analysis. Participants were rewarded based on the number of questionnaires completed, not directly compensated. In this study we only analyzed the data from the DCE part.

###  Exclusion Criteria

 Participants were excluded if the interview was not completed, completed DCE data in less than 45 seconds, and because of potentially problematic data, including respondents who gave a suspect response pattern (AAAAAAAAAA, BBBBBBBBBB, ABABABABAB, and BABABABABA), and responses that were inconsistent between the two designs presented below.

###  Elicitation Tasks Design

**Figure 1:**
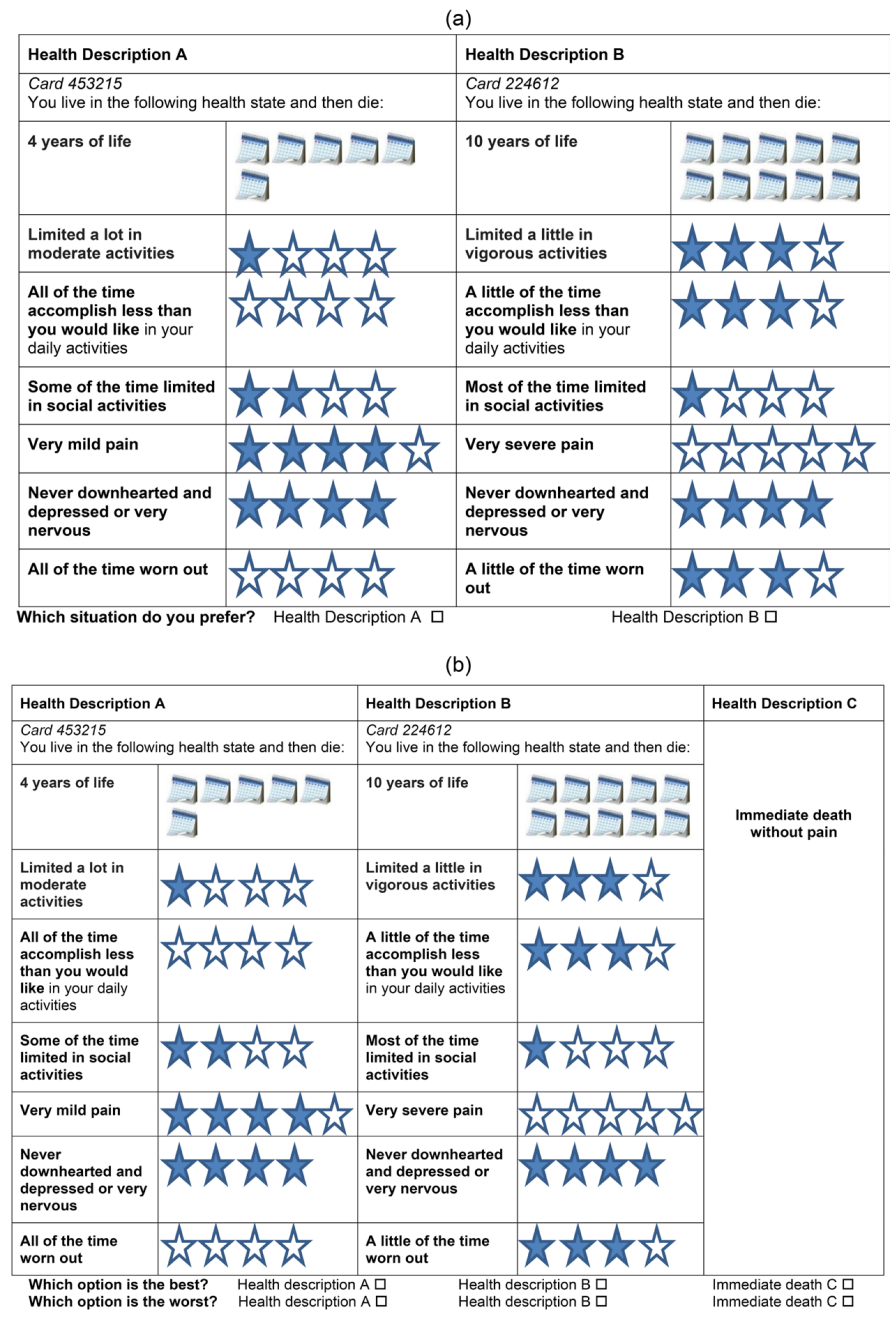
(a) Example of a Discrete Choice Experiment With Time Duration. (b) Example of Best-Worst Scale With Duration Choice Task.

 After introducing the research objectives, respondents were requested to provide socio-demographic information and to self-assess their health state using the SF-6Dv2. DCE_TTO_ elicitation tasks were implemented in two designs: binary choice sets (Design 1, see Figure 1a) and best-worst choice sets (Design 2, see Figure 1b). Participants completed both designs. Firstly, they were presented with binary choice sets and an associated duration, where duration refers to life-years (ie, Design 1). Following that, the participants were presented with three triple choices, which consisted of binary choice sets along with a third scenario describing “immediate death” (ie, Design 2). In this design, participants were asked to indicate which of the three choices—choice A, B, or C (immediate death)—was the best and which was the worst. The survival duration levels of 1, 4, 7, and 10 years were included alongside the health state dimensions for the scenarios, as they have been effectively employed in previous DCE_TTO_ valuation surveys of the SF-6D valuation.^10-13^ The maximum duration of ten years was employed to align with the time horizon commonly used in TTO. One year was set as the minimum unit to represent a full year, while 4 and 7 years were included within the range of 1 to 10. The variation in intervals allowed us to present a variety of duration ratios in combination with health state dimensions.

###  Experimental Design

 The health states for this study were selected using an efficient design procedure, which minimizes the D-error. Briefly, 120 health states were generated randomly by computer and then paired (60 pairs) based on near orthogonal arrays using SAS software. In the experimental design, both the main effects and two-way interactions between the levels of each dimension and durations were taken into account.

 To ensure robust model estimation, it is expected that a minimum of 15 observations to be collected for each pair of DCE_TTO_ tasks.^18^ This means that 900 respondents will be sufficient. However, we also needed the sample to be representative of the general population with a confidence level of 95% and a margin of error of 3%. To avoid any inefficiency related to the sample size and to ensure representativity, the minimum total target sample size was thus set at 1067. Indeed, participants were randomly assigned to 13 choice sets. The first 10 were administered using the Design 1 task format, and the last three choice sets were conducted using the Design 2 format. Design 1 included a total of 60 choice sets (ie, six blocks of ten pairs), which were selected from all combinations of SF-6Dv2 health states and duration as described above. The same 60 choice sets were also used in Design 2 along with a third scenario describing immediate death. For each respondent, the 3 tasks to complete in Design 2 were randomly selected from the block of ten pairs in which they were previously allocated. The choice sets in Design 1 were consistently presented first, followed by Design 2. This sequential order allowed for a gradual increase in task complexity, moving from presenting pairs to presenting triplets.

###  Modeling 

 Conditional logit regression was the most commonly used model for the DCE tasks, following the approach proposed by Bansback et al.^19^

*U*_ij_ = *αTIME*_ij_ + *βX*_ij_*TIME*_ij_ + *ε*_ij_

 Where *U*_ij_ represents the utility of option j in a choice set for respondent i; *TIME*_ij_ represents the survival duration; *α *represents the coefficient for the survival duration; *X*_ij_*TIME*_ij_ denotes the interactions between dimension levels and survival duration; *β* represents the coefficients for interaction terms, and *ε*_ij_ accounts for the error term. The independent variables consisted of a duration parameter and 25 parameters derived from the health state dimensions, with the exception of PA, which was represented by six dummy variables, all other dimensions were coded using five dummy variables with a baseline level of 1 indicating no problem. The model calculates parameters to capture the interactions between levels of each dimension (excluding the best level) and duration. Furthermore, the model also estimates a specific value for the continuous duration variable, enabling a comprehensive analysis of the relationships between dimension levels and duration. Duration was modeled as a linear and continuous variable. This assumption was examined by modelling duration as a categorical variable and plotting the duration coefficients. The plot of duration coefficients showed a consistent linear trend, thereby supporting the initial assumption of linearity and continuity (Supplementary file 1, Figure S1). The model is referred to as the unanchored model. In this model, the estimates for dimension level interactions are initially unanchored, which means they are not directly mapped onto the full health–dead utility scale. However, to anchor these estimates onto the utility scale, the estimates for health state and duration interactions are divided by the estimated coefficient for duration. This anchoring process ensures that the dimension level interactions are aligned with the full health–dead utility scale.^19^

###  Model Evaluation 

 Several models were tested for the value set. First, the Design 1 data were exclusively modeled and generated Model 1, which exhibited inconsistencies within the model. Inconsistent models do not enforce the a priori ordering of dimension levels, which means they can include disordered coefficients. In such cases, an increase in health severity may lead to an increase in utility, instead of the expected decrease. Subsequently, the disordered dimension levels of Model 1 were merged with adjacent levels to create a consistent model, referred to as Model 2 (ie, parsimonious model). Second, both Design 1 and Design 2 data were used. Model 3 and Model 4, respectively included inconsistent and consistent parameters. We proceeded from Model 4 to Model 5 to examine the addition of an interaction term, which was added if any of the dimensions were at the worst level (known as the “WORST” term). This addition provides a general estimate for the presence of poor health at any level. To analyze the combination of Design 1 and 2 data, a duration of zero was assigned to the “dead” option. This was done to convert the tripled tasks to binary format and subsequently analyze them as binary. In other words, stated preferences were analyzed through three pairwise choices: A vs B, A vs C (dead), B vs C (dead).

 The results are presented in two forms: the unanchored estimates, which are on a latent scale and thus not directly comparable across models in terms of magnitude, and the anchored estimates, which are on a scale ranging from 1 for full health to 0 for dead, allowing for comparisons across models. The estimates are anchored using the marginal rate of substitution, which is calculated by dividing the coefficient for each level of each attribute by the coefficient for duration. Standard errors and confidence intervals of the QALY estimates of the anchored Models were calculated using the ‘‘wtp’’ command in STATA 16, with the default delta method.^20^

 Finally, the performance of candidate models for the value set was assessed based on several criteria: (1) the number of logically consistent parameters which focuses on the monotonicity of model parameters. Theoretically, estimated coefficients for logically worse health states should have lower magnitudes than those for logically better health states within each dimension; (2) the number of significant levels; (3) the magnitude of coefficient decrements for different dimensions and levels; (4) magnitudes of standard errors; (5) overall utility ranges; (6) percentage of states worse than dead (WTD). We evaluated the fit of a statistical model by utilizing commonly used fit statistics, namely the log likelihood and the Bayesian information criterion. These statistics offer valuable insights into the extent to which the model aligns with the observed data, while considering the complexity of the model.

###  Evaluating Heterogeneity

 Conditional logit assumes that all respondents share a common unobservable set of values, which may not be realistic given the varying perceptions of health among individuals. To address this limitation, we employed mixed logit modeling to investigate preference heterogeneity. To run the mixed logit model, it is required to enhance the STATA software because it is actually unable to run mixed logit with more than 20 random parameters. A detailed description of this improvement process can be found in Supplementary file 2. Mixed logit modeling enables the estimation of both heterogeneous and homogeneous parameters.^21^ The model can be represented by equation (1), which expresses the utility for individual *i* associated with choice *j *in scenario *s* as follows:

*u*_ijs_*= β*x´_ijs_ + (ƞ_i_x´_ijs_ + *ε*_ijs_)

 In this equation, *β* represents the coefficient vector, x´_ijs_ is the explanatory variable vector, and ƞ_i_ is the variability term. The term ƞ_i_x´_ijs_ captures the heterogeneity by allowing for variation in the coefficients across individuals, while *ε*_ijs_ represents the error term. The presence of significant standard deviations for any parameter implies the existence of heterogeneity in preferences among respondents. In the examined model (Model 6), both the dimension × duration parameters and individual duration parameters were treated as heterogeneous and independent of each other.

## Results

 Of the 5028 subjects who were invited in 2016 and 2018, 1979 subjects were randomly selected to perform DCE tasks, and 1208 completed until the end. We excluded 55 participants due to suspected response patterns and completion in times less than 45 seconds. Out of those who completed tasks and were included for analysis, a total of 22 960 observations for Design 1 and 43 814 for both Design 1 and 2 data were reached. Table 1 presents a comprehensive overview of the sample demographics, along with a comparative analysis with the demographics of the general population of Quebec. In general, the sample was representative of the general population with respect to gender, mean age, marital status, and occupation. However, the sample displayed a higher level of education and a lower proportion residing in urban area when compared to the overall population. Table 1 also shows a significant difference in the characteristics of age, marital status, education level, and occupational status between those who fully completed the DCE tasks and those who did not. The subjects who did not fully complete the DCE were observed to have lower levels of education, living alone, older, and retired. The mean ± standard deviation (SD) time spent for all choice sets was 7.82 ± 7.67 minutes.

**Table 1:** Characteristics of the Study Sample Compared to the Quebec General Population, and Characteristics of Respondents With Complete and Incomplete Discrete Choice Experiment Tasks

**Characteristics**	**Study Sample ** **(n = 1153)**		**Quebec Population**^a^	**Sample Completed ** **(n = 1208)**		**Sample Incomplete** **(n = 771)**		***P*** **Value**^b^
	**N**	**%**	**%**	**N**	**%**	**N**	**%**	
Gender								
Male	466	40.42	49.4	491	40.65	311	41.08	.848
Female	687	59.58	50.6	717	59.35	446	58.92	
Age (y)								
18-24	80	6.94	10.2	80	6.62	22	2.85	<.0001
25-34	174	15.09	16.2	182	15.07	47	6.10	
35-44	199	17.26	16.7	210	17.38	76	9.86	
45-54	284	24.63	16.6	296	24.50	144	18.68	
55-64	259	22.46	17.7	275	22.76	231	29.96	
65+	157	13.62	22.7	165	13.66	251	32.56	
Mean	47.76		49.3	47.8		56.29		
Marital status								
Married/living with partner	656	56.90	56.3	684	56.62	405	55.10	<.0001
Single	324	28.10	29.4	344	28.48	162	22.04	
Divorced/separated	142	12.32	8.6	147	12.17	131	17.82	
Widowed	31	2.69	5.7	33	2.73	37	5.03	
Education level								
Secondary or less	288	24.98	39.2	291	24.09	237	32.24	.001
Professional diploma	171	14.83	17.6	164	13.58	105	14.29	
CEGEP	360	31.22	18.2	378	31.29	219	29.80	
Baccalaureate	265	22.98	17.4	294	24.34	144	19.59	
Master	59	5.12	6.8	70	5.79	29	3.95	
University doctorate	10	0.87	0.7	11	0.91	1	0.14	
Occupational status								
Employee or self-employed	587	50.91	59.5	627	51.90	293	39.54	<.0001
Retirement	280	24.28	27.4	293	24.25	315	42.51	
Student	84	7.29	3.3	89	7.37	25	3.37	
At home	81	7.03		92	7.62	51	6.88	
Unemployed	74	6.42	7.2	82	6.79	37	4.99	
Sick leave	47	4.08	2.5	25	2.07	20	2.70	
Area								
Urban	780	67.65	80.6	815	67.47	473	68.35	.691
Rural	373	32.35	19.4	393	32.53	219	31.65	

Abbreviation: CEGEP, Collège d›enseignement général et professionnel. ^a^ General population aged 18 years or older; ^b^ Chi-square test.

###  Distribution of Responses on SF-6Dv2 Dimensions

**Figure 2:**
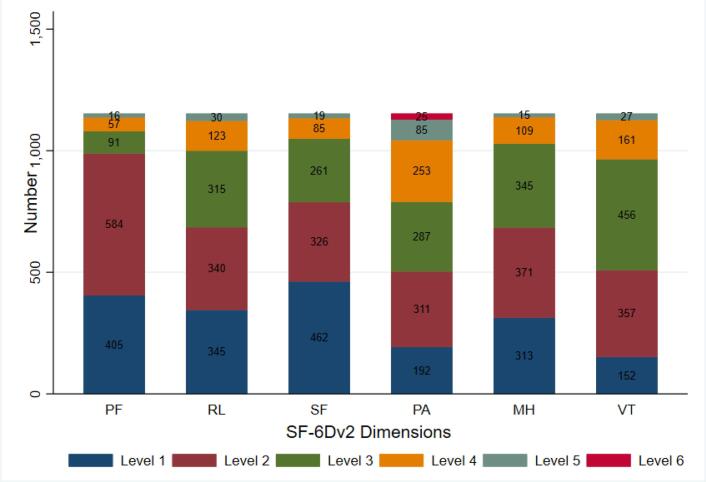
Distribution of the Reported Problems by Participants(level 6 only available for Pain). Abbreviations: SF-6Dv2, new version of Short-Form 6-Dimension; PF, physical functioning; RL, role limitations; SF, social functioning; PA, pain; MH, mental health; VT, vitality.

 The problems reported by participants on the SF-6Dv2 dimensions with respect to their level are displayed in Figure 2. The number of respondents reporting “level 1” on the dimensions of PF, RL, SF, PA, MH, and VT were 405, 345, 462, 192, 313, and 152 cases, respectively. Health problems were most frequently reported in the VT dimension (86.82%), while the SF dimension had the lowest frequency of reported health problems (59.93%). The distribution pattern of problems among the entire sample that successfully completed the SF-6Dv2 questionnaire exhibited a consistent similarity (Figure S2).

###  Estimation of Unanchored Models

 Unanchored Modeling results of the DCE tasks are presented in Table 2. The coefficients generated by the models are latent values that are not on a 0 to 1 scale; therefore, they cannot directly be used to estimate QALYs. As expected, the estimates for duration in all models are positive, indicating a preference for longer durations of living. Model 1 from Design 1 data shows that all levels in each dimension are ordered except for levels 3 and 4 of RL, and most estimations are statistically significant at level <.001. Model 2 is a parsimonious consistent model, where levels 3 and 4 of RL were aggregated.

**Table 2:** Unanchored Models

	**Model 1 (Inconsistent)**		**Model 2 (Consistent)**		**Model 3 (Inconsistent)**		**Model 4 (Consistent)**		**Model 5 (Consistent)**		**Model 6(Heterogeneity)**	
	**β**	**SE**	**β**	**SE**	**β**	**SE**	**β**	**SE**	**β**	**SE**	**β (SE)**	**SD**
PF2×LY	-0.035**	0.007	-0.033**	0.007	-0.038**	0.007	-0.034**	0.007	-0.042**	0.007	-0.039 (0.008)**	0.022
PF3×LY	-0.047**	0.008	-0.047**	0.008	-0.053**	0.007	-0.050**	0.007	-0.051**	0.007	-0.052 (0.009)**	0.066**
PF4×LY	-0.076**	0.008	-0.075**	0.008	-0.076**	0.007	-0.074**	0.007	-0.079**	0.007	-0.090 (0.009)**	0.068**
PF5×LY	-0.081**	0.009	-0.080**	0.009	-0.113**	0.007	-0.111**	0.007	-0.091**	0.008	-0.130 (0.010)**	0.060**
RL2×LY	-0.028*	0.008	-0.017*	0.005	-0.038**	0.007	-0.019**	0.005	-0.027**	0.005	-0.047 (0.009)**	0.072**
**RL3×LY**	-0.025*	**0.007**	-0.017*	0.006	-0.040**	0.006	-0.026**	0.005	-0.030**	0.005	-0.050 (0.008)**	0.040*
**RL4×LY**	**-0.014**	**0.008**	-0.017**	0.006	-0.026**	**0.006**	-0.026**	0.005	-0.030**	0.005	-0.030 (0.009)**	0.069**
RL5×LY	-0.104**	0.008	-0.095**	0.006	-0.094**	0.007	-0.075**	0.005	-0.058**	0.005	-0.122 (0.009)**	0.072**
SF2×LY	-0.023*	0.007	-0.021*	0.007	-0.016*	0.006	-0.012*	0.006	-0.014*	0.006	-0.032 (0.008)**	0.025
SF3×LY	-0.024**	0.007	-0.023*	0.007	-0.025**	0.006	-0.023**	0.006	-0.027**	0.006	-0.032 (0.008)**	0.069**
SF4×LY	-0.039**	0.007	-0.041**	0.007	-0.042**	0.006	-0.044*	0.006	-0.051**	0.006	-0.057 (0.008)**	0.059**
SF5×LY	-0.068**	0.008	-0.069**	0.008	-0.080**	0.006	-0.081**	0.007	-0.066**	0.007	-0.104 (0.008)**	0.058**
PA2×LY	-0.023**	0.008	-0.027**	0.007	-0.026**	0.006	-0.032**	0.006	-0.026**	0.006	-0.029 (0.008)*	0.017
PA3×LY	-0.050**	0.008	-0.056**	0.008	-0.035**	0.007	-0.045**	0.007	-0.036**	0.007	-0.038 (0.010)**	0.065**
PA4×LY	-0.068**	0.008	-0.071**	0.008	-0.052**	0.006	-0.057**	0.006	-0.062**	0.006	-0.062 (0.009)**	0.059**
PA5×LY	-0.077**	0.010	-0.081**	0.010	-0.074**	0.008	-0.083**	0.008	-0.076**	0.008	-0.086 (0.011)**	0.063**
PA6×LY	-0.111**	0.009	-0.117**	0.008	-0.126**	0.007	-0.137**	0.007	-0.114**	0.008	-0.162 (0.011)**	0.112**
MH2×LY	-0.021**	0.007	-0.019**	0.007	-0.013*	0.005	-0.011*	0.005	-0.022**	0.006	-0.030 (0.008)**	0.045**
MH3×LY	-0.046**	0.008	-0.045**	0.008	-0.039**	0.007	-0.037**	0.007	-0.056**	0.007	-0.066 (0.009)**	0.054**
MH4×LY	-0.062**	0.007	-0.060**	0.007	-0.049**	0.006	-0.047**	0.006	-0.057	0.006	-0.070 (0.008)**	0.058**
MH5×LY	-0.064**	0.007	-0.062**	0.007	-0.085**	0.006	-0.083**	0.006	-0.067**	0.006	-0.115 (0.008)**	0.050**
VT2×LY	-0.020*	0.006	-0.020**	0.006	-0.013*	0.005	-0.011*	0.005	-0.012*	0.005	-0.014 (0.007)*	0.053**
VT3×LY	-0.037**	0.007	-0.03**	0.006	-0.022**	0.005	-0.021**	0.005	-0.021**	0.005	-0.030 (0.007)**	0.075**
VT4×LY	-0.048**	0.007	-0.047**	0.007	-0.048**	0.006	-0.046**	0.006	-0.040**	0.006	-0.052 (0.008)**	0.036
VT5×LY	-0.098**	0.007	-0.101**	0.007	-0.062**	0.006	-0.066**	0.005	-0.054**	0.006	-0.081 (0.008)**	0.076**
LY	0.255**	0.013	0.248*	0.012	0.342**	0.011	0.328**	0.011	0.356**	0.012	0.429 (0.015)**	0.004
WORST×LY									-0.052**	0.008		
#illogically ordered	2		0		1		0		0		0	
#non-significant	1		0		0		0		0		0	
# observations	22 960		22 960		43 814		43 814		43 814		43 814	
Log likelihood	-7281		-7283		-13 441		-13 449		-13 430		-13 294	
BIC	14 824		14 817		27 161		27 167		27 138		26 410	

Abbreviations: SE, standard error; SD, standard deviation; PF, physical functioning; RL, role limitations; SF, social functioning; PA, pain; MH, mental health; VT, vitality; BIC, Bayesian information criterion; LY, life-years. Models 1 and 2 are for Design 1; Models 3 and 4 are for Design 1 + Design 2; Model 5 is the same as Model 4 + WORST×LY; Model 6 is a mixed logit model. **Bold **indicate disordered estimates; * Significant at.05; ** Significant at <.001.

 Model 3, including both Design 1 and 2 data, showed only a disorder for level 3 of RL, while almost all levels of dimensions were significant at <.001, except for level 2 of SF, MH, and VT (*P* < .05). A parsimonious consistent model was constructed in Model 4, where levels 3 and 4 of RL were aggregated. Model 5 included any additional effect from dimensions at the most severe levels (WORST) and the order of estimates consistently remained. The negative sign associated with the WORST term reflects a further reduction in utility when a state includes at least one dimension at the most severe level. Furthermore, the inclusion of the WORST term has an additional effect of diminishing the coefficient estimates associated with the most severe level across all six dimensions when compared to Model 4. This implies that experiencing a severe health problem in any specific area has a global impact on overall health while diminishing the impact of each dimension individually.

###  Estimation of Anchored Models 

Table 3 showed the anchored models obtained from Model 2, 4, and 5. The coefficients generated by the models can be used to derive a SF-6Dv2 value set and enabling comparisons across different models. For all dimensions, the lower severity levels of Model 5 show a smaller reduction compared to the other Models. However, Model 5 shows a larger reduction for most dimension levels compared to the others. The anchored Model 2 generates values from 1 to -1.114, and 39.01% of all 18 750 states are negative in this model. Model 4 reveals that introducing the Design 2 choice sets reduces the utility range (1 to -0.693) and the percentage of negative states (to 13.01%). The level coefficients of Model 5, which are generally smaller than those of Model 4, include an additional decrement due to the WORST term. This leads to a narrower utility range (1 to -0.407) with a percentage WTD of 13.32%. Model 5, with four non-significant parameters, indicated a higher count of non-significant parameters compared to the other models. The ranking of importance, based on the overall dimension magnitude as a proxy for importance, also shows some changes across the models. It is PA, VT, RL, PF, SF, and MH in model 2, while this order remains consistent across models 4 and 5 with PA, PF, MH, SF, RL, and VT. The largest decrements in level 6 for the PA dimension was found in all models.

**Table 3:** Anchored Models

	**Model 2**			**Model 4**			**Model 5**		
	**β**	**SE**	**95% CI**	**β**	**SE**	**95% CI**	**β**	**SE**	**95% CI**
PF2	-0.133**	0.029	(-0.193, -0.077)	-0.103**	0.020	(-0.146, -0.065)	-0.117**	0.037	(-0.157, -0.083)
PF3	-0.189**	0.030	(-0.250, -0.132)	-0.152**	0.020	(-0.195, -0.114)	-0.143**	0.037	(-0.180, -0.106)
PF4	-0.303**	0.031	(-0.365, -0.240)	-0.225**	0.020	(-0.266, -0.185)	-0.221**	0.038	(-0.261, -0.186)
PF5	-0.323**	0.033	(-0.391, -0.259)	-0.338**	0.021	(-0.382, -0.298)	-0.255**	0.046	(-0.303, -0.211)
RL2	-0.068**	0.023	(-0.117, -0.025)	-0.057**	0.015	(-0.088, -0.028)	-0.075*	0.029	(-0.106, -0.049)
RL3	-0.068**	0.024	(-0.120, -0.023)	-0.079**	0.015	(-0.110, - 0.050)	-0.084**	0.028	(-0.111, -0.056)
RL4	-0.068**	0.024	(-0.120, -0.023)	-0.079**	0.015	(-0.110, -0.050)	-0.084**	0.028	(-0.111, -0.056)
RL5	-0.383**	0.027	(-0.438, -0.329)	-0.228**	0.015	(-0.261, 0.201)	-0.162**	0.033	(-0.197, -0.131)
SF2	-0.084*	0.030	(-0.146, -0.027)	-0.036	0.019	(-0.077, 0.002)	-0.039	0.035	(-0.076, -0.006)
SF3	-0.092**	0.029	(-0.153, -0.037)	-0.070**	0.019	(-0.109, -0.034)	-0.075*	0.036	(-0.111, -0.040)
SF4	-0.165**	0.028	(-0.224, -0.112)	-0.134**	0.018	(-0.171, -0.100)	-0.143**	0.033	(-0.176, -0.110)
SF5	-0.278**	0.032	(-0.342, -0.215)	-0.246**	0.020	(-0.287, -0.207)	-0.185**	0.042	(-0.227, -0.144)
PA2	-0.109**	0.030	(-0.170, -0.051)	-0.097**	0.018	(-0.136, - 0.062)	-0.073*	0.035	(-0.108, -0.038)
PA3	-0.226**	0.032	(-0.292, -0.166)	-0.137**	0.020	(-0.179, -0.099)	-0.101*	0.039	(-0.140, -0.063)
PA4	-0.286**	0.031	(-0.350, -0.227)	-0.173**	0.019	(-0.213, -0.136)	-0.174**	0.036	(-0.210, -0.139)
PA5	-0.327**	0.042	(-0.413, -0.247)	-0.253**	0.024	(-0.302, -0.207)	-0.213**	0.046	(-0.259, -0.167)
PA6	-0.472**	0.038	(-0.549, -0.399)	-0.417**	0.023	(-0.465, -0.372)	-0.320**	0.052	(-0.373, -0.271)
MH2	-0.076*	0.029	(-0.137, -0.023)	-0.033*	0.016	(-0.070, -0.001)	-0.061	0.033	(-0.095, -0.029)
MH3	-0.181**	0.030	(-0.245, -0.124)	-0.112**	0.021	(-0.156, -0.072)	-0.157**	0.040	(-0.199, -0.120)
MH4	-0.242**	0.030	(-0.305, -0.186)	-0.143**	0.018	(-0.179, -0.107)	-0.160**	0.034	(-0.194, -0.127)
MH5	-0.250**	0.028	(-0.310, -0.198)	-0.253**	0.017	(-0.289, -0.219)	-0.188**	0.038	(-0.226, -0.150)
VT2	-0.080**	0.025	(-0.133, -0.034)	-0.033*	0.016	(-0.068, -0.004)	-0.033	0.030	(-0.065, -0.005)
VT3	-0.145**	0.025	(-0.199, -0.098)	-0.064**	0.017	(-0.100, -0.033)	-0.058	0.031	(-0.090, -0.028)
VT4	-0.189**	0.027	(-0.246, -0.137)	-0.140**	0.017	(-0.177, -0.107)	-0.112**	0.034	(-0.148, -0.081)
VT5	-0.408**	0.032	(-0.472, -0.344)	-0.201**	0.017	(-0.236, -0.167)	-0.151**	0.035	(-0.187, -0.117)
WORST							-0.146**	0.045	(-0.190, -0.101)
U(555655)	-1.114			-0.683			-0.407		
WTD (%)	39.01			13.01			13.32		
#non-significant	0			1			4		
Order in coefficient magnitude	PA-VT-RL-PF-SF-MH			PA-PF-MH-SF-RL-VT					

Abbreviations: CI, confidence interval; SE, standard error; PF, physical functioning; RL, role limitations; SF, social functioning; PA, pain; MH, mental health; VT, vitality; WTD, worse than dead. Model 2 is for Design 1; Model 4 is for Design 1 + Design 2; Model 5 is similar to Model 4 + WORST. * Significant at.05; ** Significant at <.001.

**Figure 3:**
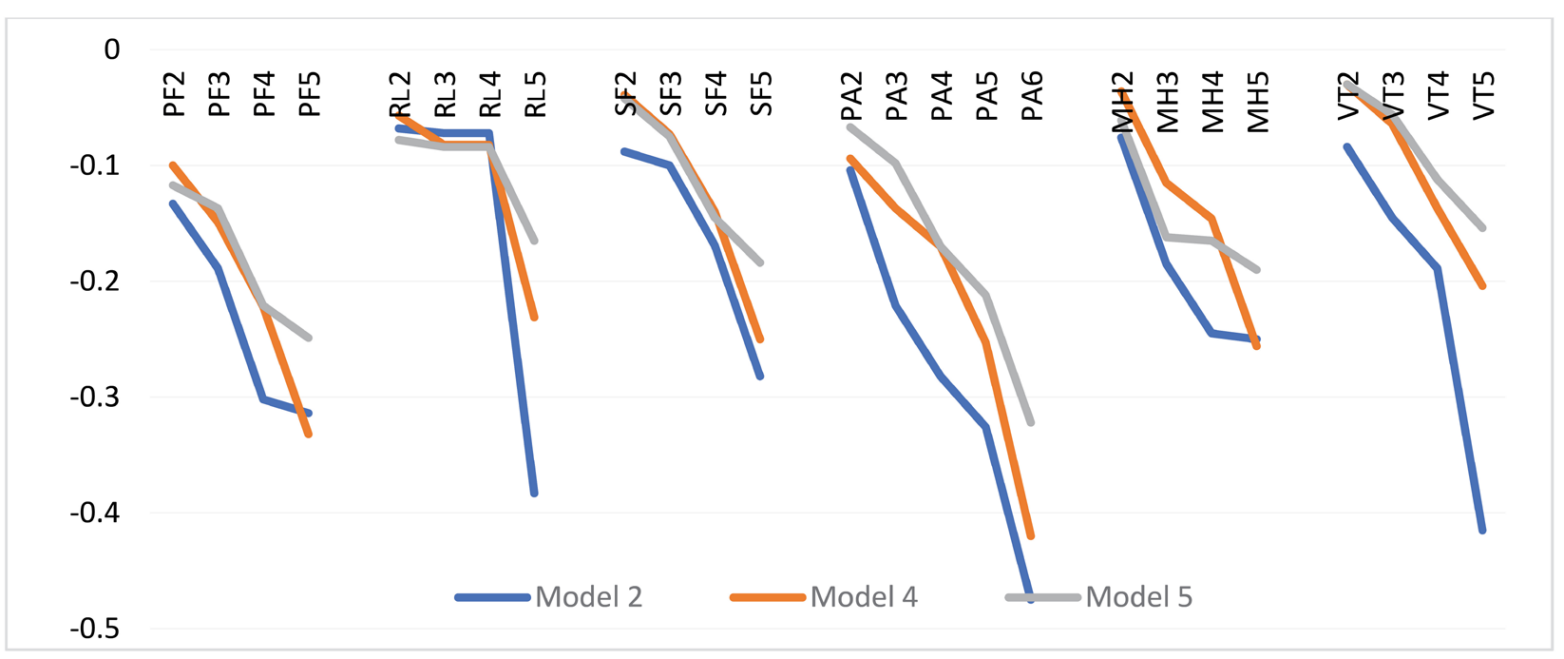
Decrements for Each Dimension of the SF-6Dv2. Abbreviations: PF, physical functioning; RL, role limitations; SF, social functioning; PA, pain; MH, mental health; VT, vitality; SF-6Dv2, new version of Short-Form 6-Dimension.

 The utility reductions generated according to each of the dimensions are visually presented in Figure 3. For most dimensions, Model 5 shows smaller decrements compared to Models 2 and 4 across various dimension levels. The only exception to this trend is observed in the lower severity levels of MH.

###  Heterogeneity Assessment

 The results of the evaluation of preference heterogeneity on Design 1 and 2 data are presented in Table 2. The standard deviations of mixed model (Model 6) show evidence of heterogeneity for all level of dimensions, except for level 2 of the PA, SF, and PF dimensions, and for level 4 of VT dimension. Moreover, this evidence is particularly strong for the most severe levels of PA, and VT. There is no significant preference heterogeneity around duration.

###  Comparison Across All Anchored Models

 As illustrated in Table 3, the PA dimension as the first order of the overall decrement of the dimensions was the same for all models. Also, all parameter estimates of models were consistent (ie, decrements are ordered). Model 2 determined 39.01% health states to be WTD health states, while in Model 4, the estimated percentage was 13.01%, and in Model 5, it was 13.32%. The utility values of the worst state 555655 were -1.114 for Model 2, whereas it was -0.683 and -0.407 for Model 4 and 5, respectively. The range of the standard errors of anchored model 4 (0.015 to 0.024) is almost half the size of those of Model 5 (0.028 to 0.052) and smaller than the standard errors of Model 2, (0.024 to 0.042). Models 4 and 5 estimated fewer WTD health states compared to Model 2 (13.01%, 13.32%, and 39.01%). Furthermore, the standard errors of anchored Model 4 are smaller than those of Model 5, indicating a higher level of precision in Model 4. Therefore, we recommend calculating the utility value of each health state obtained from the SF-6Dv2 using Model 4. This model is provided in Excel format in Supplementary file 3, so that it can be used easily. In general, the model constructed using Design 1 and 2 data generated a narrower value set compared to Model 2, which was constructed using Design 1 data alone.

 The final model is used to calculate the utility value of each health state (Hi) as follows:

 U(Hi) = 1-(0.103PF2 + 0.152PF3 + 0.225PF4 + 0.338PF5 +0.057RL2 + 0.079RL3 + 0.079RL4 + 0.228RL5 + 0.036SF2 + 0.070SF3 + 0.134SF4 + 0.246SF5 + 0.097PA2 + 0.137PA3 + 0.173 PA4 + 0.253PA5 + 0.417PA6 + 0.033MH2 + 0.112MH3 + 0.143MH4 + 0.253MH5 + 0.033VT2 + 0.064VT3 + 0.140VT4 + 0.201VT5)

 For example, the utility value of state 223221 (ie, I am a little limited in vigorous activities, I accomplish less than you would like a little of the time, my social activities are limited some of the time, I am depressed or very nervous a little of the time, and I am not worn out at all) is calculated as follows:

 1-(0.103 + 0.057 + 0.070 + 0.097 + 0.033+0) = 0.64

## Discussion

 Since there are differences in health preferences due to disparities in culture, economy, or other socioeconomic factors across countries, the use of alternative value sets is required. Therefore, this study presents health preferences and a value set derived from the adult Quebec general population for the last version of the SF-6D (SF-6Dv2). The SF-6Dv2 is an updated and improved version of the SF-6D, designed for calculating QALYs in economic evaluations and assessing HRQoL. To derive values attached to the 18,750 SF-6Dv2 health states, an online survey was administered with 1208 participants in the DCE_TTO_ elicitation tasks divided in two designs: binary choice sets and triple choice sets. The sociodemographic characteristics of the respondents included (n = 1153) closely matched those of the general population in terms of gender, mean age, marital status, and occupation. This characteristic makes SF-6Dv2 suitable for health economic evaluations, which can contribute to local healthcare financing following the principles of HTA.^5^

 The analysis of problem distribution on levels of each SF-6Dv2 dimension in both the sample study and the completed SF-6Dv2 sample consistently revealed that the VT dimension had the highest frequency of reported health problems, while the SF dimension had the lowest. Similar findings were obtained in the United Kingdom during the estimation of a value set for SF-6Dv2.^10^ The highest frequency of the problems on the VT was also reported in China, while the lowest frequency of problems was reported on the PF.^12^ The relatively large proportion of urban population in the sample of China (59.6%) might be contributing to the observed similarities in reported health problems on the VT dimension with Anglo-Saxon countries. Urban populations often share similar lifestyle factors such as sedentary habits, high work-related stress, and increased exposure to environmental pollutants, which can influence VT. The second highest frequency of reported health problems is observed in the PA dimension in both our study and the UK^10^, while it was in MH for China^12^. These similarities between two countries of the western world and their dissimilarities with China could be explained by cultural differences. Our findings also align with those of other studies revealing the reports of level 6 in the PA dimension.^10-13^ This suggests that the new version of SF-6Dv2 demonstrates good discriminatory ability.

 The value set generated in Model 4 was preferred as this model has monotonicity statistical significance of the coefficients and the highest precision of the model coefficients (ie, the lowest standard errors). Meanwhile, this model differs from Model 2 in terms of %WTD, value range, and order in coefficient. These differences can be explained by the different designs used for the two models. When comparing the SF-6Dv2 value set of model 4 with the Australian value set, the range of values was found to be similar, despite using different health states for the DCE_TTO_ approach. Specifically, the range of values was from 1 (111111) to -0.683 (555655) for our study and from 1 (111111) to -0.685 (555655) for Australia,^11^ while these ranges are different from 1 (111111) to -0.535 (555655) for China,^12^ from 1 (111111) to -0.796 (555655) for Iran,^13^ and from 1 (111111) to -0.574 (555655) for the United Kingdom,^10^ with both the United Kingdom and China value sets generating higher values. A part of the similarity between our value set and Australia can be explained by the fact that both countries share common cultural influences and societal norms. Moreover, both Canada and Australia are known for their diverse and multicultural societies. This diversity has likely influenced the values and priorities of their populations, leading to certain similarities in reported health problems and experiences related to the assessment of health profiles.

 Although PA dimension had the largest decrement in the results, the order of other dimensions between the two designs was not similar. This change may be explained by the addition of a third scenario describing immediate death to Design 2. These changes between models can also be observed in studies conducted in China and other studies carried out in Quebec involving patients with cancer.^12,14^ However, this study revealed that the PA dimension had the largest decrement, and VT had the smallest in the preferred model, indicating that the general population respectively gave the highest and the lowest weight to these two dimensions than other dimensions in SF-6Dv2. PA as the most important dimension in decreasing utility for SF-6Dv2 value sets was also found in people with food allergy and patients with cancer in two Canadian samples, where most respondents were also from Quebec and responded in French, while the RL was revealed as the lowest important dimension for those people.^14,15^ This small difference for RL (ranked 6 instead of 5 in our study) and VT (ranked 4 instead of 6 in our study) can be due to differences in the target population between these studies. Our findings are consistent with the highest and lowest weights of dimensions obtained from Australia value set.^11^ Pain with the highest weight was also observed in another value sets estimated so far for SF-6Dv2 (ie, the UK and China), while the dimension with the lowest importance was RL in those.^10,12^ The largest decrements in the PA dimension for SF-6Dv1 value sets were observed in all Anglo-Saxon countries and China, while such a finding was not reported in any of the non-Anglo-Saxon countries, including Hong Kong, Japan, Portugal, Brazil, Spain, and Lebanon.^8^ The ranking of the dimensions thus demonstrates both similarities and distinctions, which can be attributed to cultural and socioeconomic factors that play a crucial role in influencing the preferences of different populations. Additionally, the differences can be explained by the variation in the valuation techniques and the different regression methods used between studies. When comparing the most important dimension with decreasing weights for SF-6Dv2 between countries, the PA dimension exhibits the largest decrements in a non-Anglo-Saxon country (ie, China). A probable reason for this could be the changes introduced in SF-6Dv2 descriptive system (ie, no value set with SF-6Dv1 exists in China while it is the only non-Anglo-Saxon country to exhibit PA as the most important dimension).^8^ Hence, it is essential to consider this factor when publishing value sets for SF-6Dv2 in other countries. However, the pain/discomfort dimension of the EQ-5D-5L showed the largest decrements in countries like the United Kingdom,^22^ the Netherlands,^23^ Germany,^24^ France,^25^ and Spain,^26^ all of which are in the western region. When comparing the importance of levels of dimensions, level 6 of PA has the largest decrements (-0.417) for utility value. This finding is supported by the other three studies conducted on SF-6Dv2 in a general population. The value of this level in Australia^11^ and the United Kingdom^10^ was respectively -0.677 and -0.620. These smaller values compared to what is reported in this study may be explained by the number and type of health states selected to estimate the value set. The design of elicitations tasks and experimental choice sets in those studies were similar and based on an international protocol.

 Similar to the study conducted in Australia, a mixed logit was used to assess preference heterogeneity in the present study. This indicates that we not only emphasized the use of logit modeling approaches for ease of value estimation in decision-making but also extended the existing evidence of preference heterogeneity by examining dimension-level differences using mixed logit. The results showed that there is evidence of heterogeneity for majority of levels, especially for the most severe levels. This evidence is consistent with that in Australia,^11^ providing additional support to the latent class evidence provided from the United Kingdom.^10^ When utilizing the value set to inform resource allocation decision-making, users ought to take into account the heterogeneity in preferences. This consideration is crucial, especially in sensitivity analyses.

 Several limitations of this study need to be noted. First, the number of health states selected for the DCE task was limited. In the present study, our procedure yielded 60 choice sets (ie, 120 health states) to derive the SF-6Dv2 value set, while in other studies,^10-13^ 300 choice sets were selected. Even although these health states were selected and paired with efficiency, international protocol now recommend 300 choice sets. This number allows for a sufficient amount of data to estimate preferences accurately while still being manageable for participants to provide their responses effectively. However, our results indicate that this limitation was of little importance when comparing to other studies. Second, assessing respondent engagement in online surveys is challenging. To mitigate this issue, we controlled for potential disengagement by excluding respondents who completed the survey too quickly as well as using other exclusion criteria (see above in Methods). Third, it could be argued that there are still statistically significant differences in the distribution of background variables in the sample analyzed in the study compared with the data provided by the sample who did not fully complete the DCE survey. Indeed, those who do not complete the DCE were older and less educated, which may limit the representativity of the value set for Quebec. However, as compared to the full sample from which the sample is extracted for this study, we noticed a very similar distribution in SF-6Dv2 levels distribution for each dimension (Supplementary file 1). This is important because it suggests that even though older and less educated individuals might not have completed the DCE, their distribution of HRQoL levels isn’t drastically different from the full sample. Given that the full sample is much more representative of the general population in Quebec, this may indicate that sample bias selection was not major here. Given that French is the predominant language spoken by most people in Quebec (ie, 95% of people speak and read in French) and is the formal language of the region, our study focused on recruiting French-speaking residents for participation in online surveys. This approach was chosen to ensure linguistic coherence and cultural relevance within the study population. However, we recognize that this recruitment strategy may introduce a bias and limit the generalizability of our findings to English-speaking populations within Quebec.

## Conclusion

 This study provided a SF-6Dv2 value set in Quebec, Canada. The SF-6Dv2 value set developed using Model 4 demonstrates robustness and precision in capturing health preferences, making it a reliable tool for decision-makers in various healthcare settings. By employing this recommended value set, decision-makers can accurately measure HRQoL and calculate QALYs, thereby facilitating informed resource allocation and policy-making processes in the field of healthcare.

## Acknowledgements

 We thank Gabin F. Morillon and Richard Norman for their advice. We also thank the Fondation de l’IUSMM for their support. TGP is member of the FRQS-funded Centre de recherche de l’IUSMM.

## Ethical issues

 All procedures involving human participants were in accordance with the ethical standards of our institutional ethics committees (Comité d’éthique de la recherche of the CIUSSS de l’Estrie–CHUS #2016-1350 and Comité d’éthique de la recherche en sciences et en santé de l’Université de Montréal #2023-445). Informed written consent was obtained from all participants.

## Conflict of interests

 Authors declare that they have no conflict of interests.

## Supplementary files

Supplementary file 1. Test for Duration Linearity and Comparison Between Samples.

Supplementary file 2. Mixed Logit Modeling.

Supplementary file 3. Calculator for SF-6Dv2.
