# Supplementary material for: Valuing SF-6Dv2 Using a Discrete Choice Experiment in a General Population in Quebec, Canada
Source: Int J Health Policy Manag. 2024 Sep 7;13:8404. doi: 10.34172/ijhpm.8404 (PMC11496749; doi:10.34172/ijhpm.8404)
Supplement: Supplementary file 1 — Test for Duration Linearity and Comparison Between Samples. [file ijhpm-13-8404-s001.pdf]

Article title: Valuing SF-6Dv2 Using a Discrete Choice Experiment in a General Population in Quebec, Canada
Journal name: International Journal of Health Policy and Management (IJHPM)
Authors' information: Hosein Ameri[1,2], Thomas G. Poder[1,2]*
[1]School of Public Health, University of Montreal, Montreal, QC, Canada.
[2]Centre de Recherche de l'IUSMM, CIUSSS de l'Est de l'Île de Montréal, Montreal, QC, Canada.
***Correspondence to:** Thomas G. Poder; Email: thomas.poder@umontreal.ca
Citation: Ameri H, Poder TG. Valuing SF-6Dv2 using a discrete choice experiment in a general population in Quebec, Canada. Int J Health Policy Manag. 2024;13:8404. doi:10.34172/ijhpm.8404

Supplementary file 1. Test for Duration Linearity and Comparison Between Samples

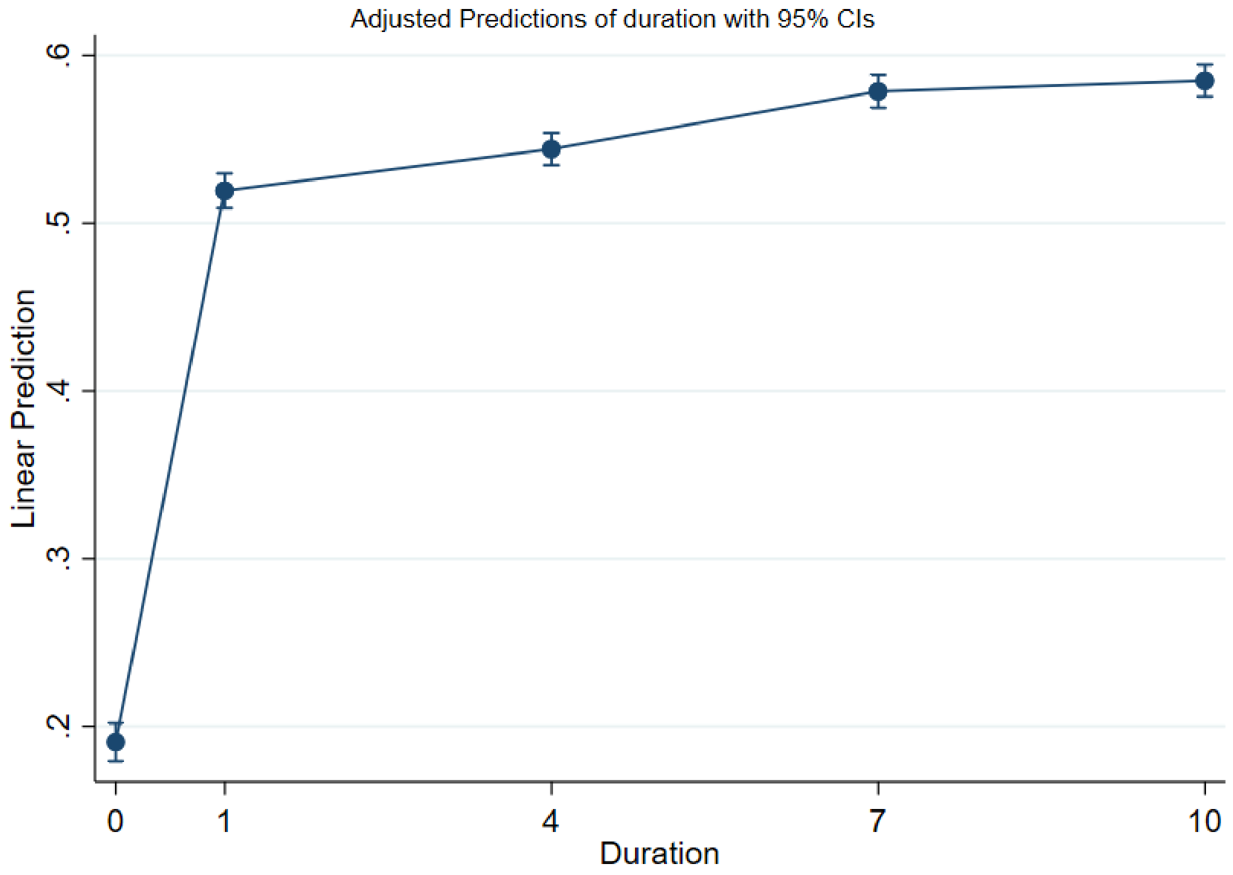

Figure S1. The plot of duration coefficients

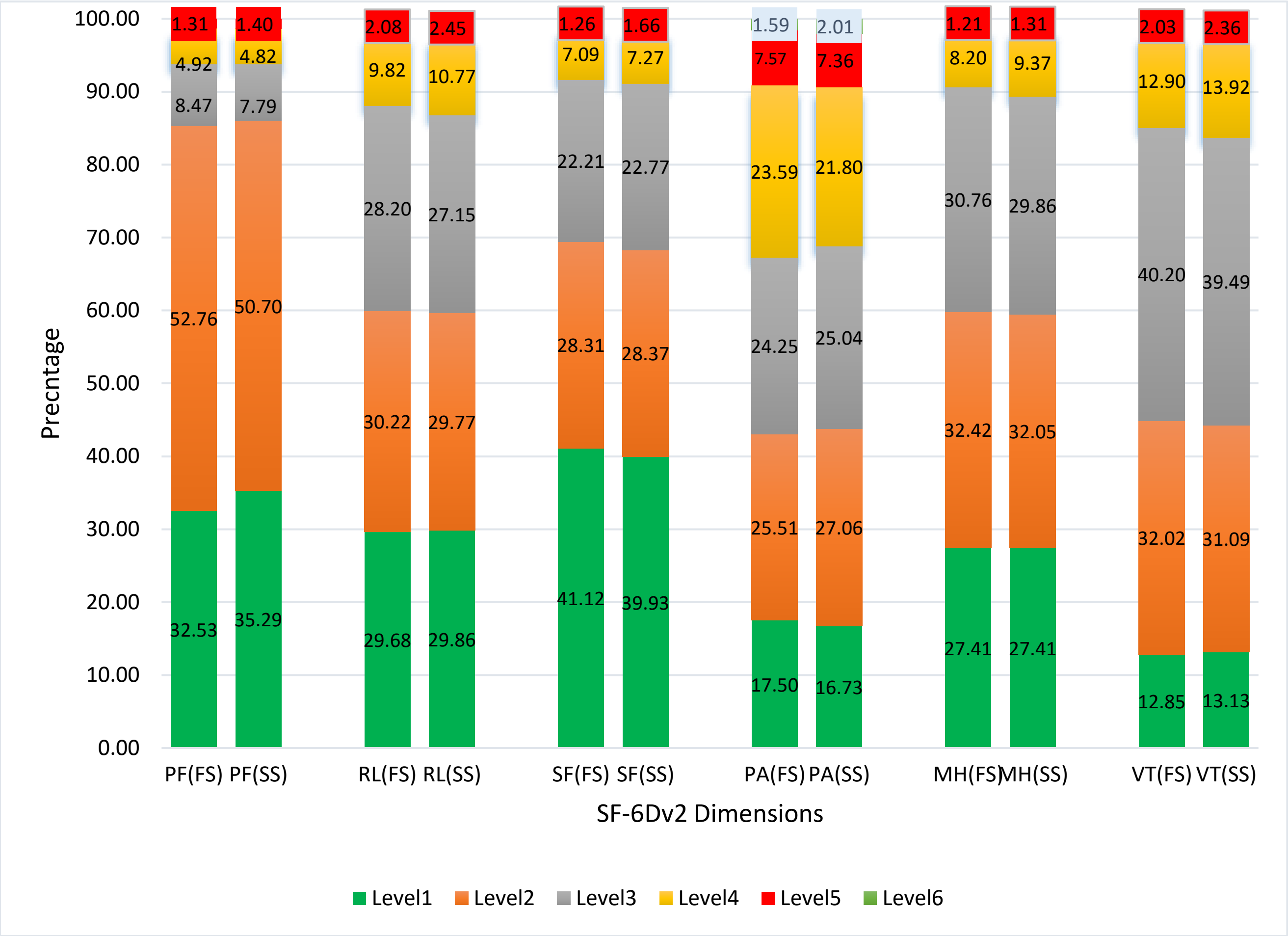

Figure S2. Distribution of the reported problems by participants in the study sample (SS) and full sample (FS) (level 6 only available for Pain)
